# Zoledronic Acid Does Not Retard Bone Union: A Randomized Controlled Trial in Fragility Intertrochanteric Femur Fractures

**DOI:** 10.7759/cureus.33948

**Published:** 2023-01-18

**Authors:** Ankit Gulia, Shakti Prasad Das, Suhas Sondur, Preethiv Rajendran, Anwesit Mohanty, Vishnu Prakash, Hari Gutta, Hirdyanshu P Pundhir, Santosh Rath

**Affiliations:** 1 Orthopaedics, Kalinga Institute of Medical Sciences, Bhubaneswar, IND; 2 Orthopaedics and Trauma, Kalinga Institute of Medical Sciences, Bhubaneswar, IND

**Keywords:** intravenous bisphosphonate, harris hip score, radiological and clinical fracture healing, intertrochanteric hip fracture, fragility fractures, zoledronic acid

## Abstract

Background: Fragility hip fracture is a leading cause of death in the elderly and is common in postmenopausal women and elderly people. In the treatment of osteoporosis, bisphosphonates (BPs) are often considered first-line medications. Zoledronic acid is the most potent and long-acting BP in clinical use and is administered as an intravenous infusion. In the context of acute fractures, the use of BPs has been controversial due to conflicting reports of their positive and negative effects on fracture healing. The purpose of this study was to determine the effect of zoledronic acid on fracture healing in intertrochanteric (IT) fragility fractures.

Methods: The study was conducted in a tertiary healthcare center after receiving scientific and ethical approval. The study included 136 patients of either gender over the age of 50 who presented with an IT femur fracture after minor trauma between November 2020 and November 2022. The total number of patients had been classified into two groups, and grouping involved random sampling: Group T (test group, n = 68; zoledronic acid injections were given on postoperative day 3) and Group C (control group, n = 68; interventions were given after fracture healing). Patients were evaluated using the Radiographic Union Score for Hip (RUSH) and Singh Index for radiological outcomes and the Harris Hip Score (HHS) for functional outcomes. The patients were followed for six months.

Results: The overall mean age was 73.25 years; in Group T, it was 72.5 ± 11.9 years, and in Group C, it was 73.7 ± 11.8 years. Of 136 patients, 69 (51%) were males and 67 (49%) were females. The average fracture healing time in Group T was 12.2 ± 3.6 weeks, while it was 13.0 ± 2.8 weeks in Group C. Functional outcomes, including HHS, were found to be better in Group T than in Group C (p < 0.005). No significant difference was observed between the two groups with respect to the radiological union, the RUSH score, or Singh Index, implying no negative effect of zoledronic acid.

Conclusions: This study demonstrates that postoperative intravenous zoledronic acid therapy does not retard fracture healing. Osteoporosis management is frequently neglected because of a surgeon's fear of ZOL retarding fracture healing and a lack of awareness among patients, resulting in low compliance. Thus, opportunistic administration of zoledronic acid postoperatively can be beneficial and will increase compliance for osteoporosis management and fracture prevention to 100%.

## Introduction

Fragility fractures following low-energy trauma are the most serious complications of osteoporosis. These fractures have a high mortality rate and have a detrimental impact on the quality of life [1]. Postmenopausal women are more likely to develop primary osteoporosis, a systemic chronic metabolic illness characterized by decreased bone mass and reduced quality [1,2]. In 2000, there were 1.5 million patients with fragility hip fractures, and by 2050, this number is expected to rise to 6.26 million [2]. The death rate increases dramatically in the five to 10 years following the occurrence of low-energy fragility hip fractures in people over 60 years [1]. After a fracture heals in a person with osteoporosis, their risk of an opposite-side fracture is three to five times greater than that of the average healthy person. The mortality rate also rises by three to four times within five years [2].

Based on the National Hip Fracture Database (NHFD), there are six best practice criteria for patients aged 60 and older with fragility hip fractures. One of the best practices is further fracture prevention through the treatment of osteoporosis [3]. According to National Institute for Health and Care Excellence (NICE) guidelines on osteoporosis and its management, oral and intravenous bisphosphonates (BPs) are cost-effective and are recommended to patients who are at increased risk of fragility fractures [4]. The pharmacology of BPs permits dosage on a weekly, monthly, intermittent, quarterly, or annual basis [5].

Medication adherence is directly related to the therapeutic regimen [5]. Better compliance with BPs is achieved with less frequent doses [6]. The injectable aminobisphosphonate zoledronic acid (ZOL) has a strong antiresorptive effect and a strong affinity for mineralizing bone [7,8]. Therefore, it is feasible that the once-yearly administration of ZOL might provide adherence to the treatment for at least 12 months and therapeutic benefit for about 15 months. It can prevent bone loss and reduce the likelihood of refracture, lowering disability and mortality rates, improving patient quality of life, and extending patients' lives.

The role of BPs in fracture healing is one of the most frequently discussed topics. Numerous researchers have investigated the role of BPs during fracture healing because it is well known that osteoclasts are important in remodeling callus formation into lamellar bone [8].

Because of conflicting reports of their positive and negative effects on fracture healing, the use of BPs has been controversial. The optimal timing of BP administration postoperatively with osteoporotic intertrochanteric femur fractures remains unknown. Solomon et al. found that using BPs in the post-fracture period was associated with a higher risk of nonunion [9]. According to Kates and Ackert-Bicknell, ZOL had a negative effect on bone healing [10]. However, in their analysis of the Health Outcomes and Reduced Incidence with Zoledronic Acid Once Yearly (HORIZON) trial data, Colón-Emeric et al. reported that there was no correlation between ZOL and delayed healing [11].

We hypothesized that intravenous ZOL does not alter fracture healing in an osteoporotic intertrochanteric fracture. The purpose of this research is to determine if ZOL alters fracture healing in intertrochanteric fragility fractures treated with proximal femoral nailing.

## Materials and methods

**Figure 1:**
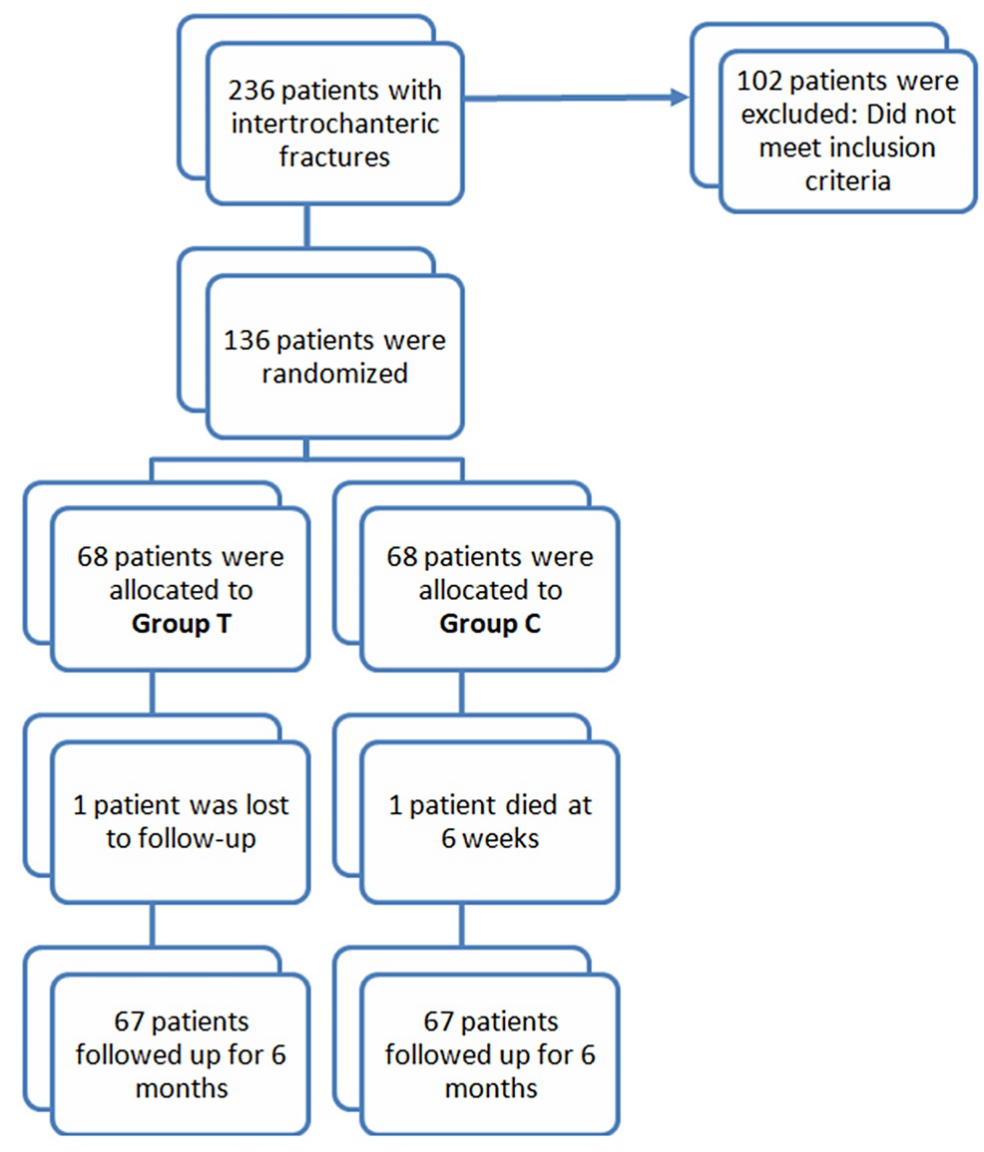
CONSORT diagram. Group T: test group, ZOL was given on postoperative day 3; Group C: control group, ZOL was given after fracture healing. ZOL: zoledronic acid.

The study was conducted in a tertiary healthcare center and has been authorized by the scientific and ethical committees with an Institutional Review Board (IRB) approval number KIIT/KIMS/IEC/488/2020 and Clinical Trials Registry-India (CTRI) trial registration number REF/2021/09/047351. The study included 136 patients of either gender over the age of 50 who presented with an intertrochanteric femur fracture after trivial trauma between November 2020 and November 2022 (Figure 1).

Patients with active malignancies, chronic metabolic disorders, polytrauma, and patients already taking BPs or oral glucocorticoids were excluded from the study (Table 1).

**Table 1:** Inclusion and exclusion criteria. ZOL: zoledronic acid.

Inclusion criteria	Exclusion criteria
Intertrochanteric fractures associated with a fall from a standing height	Patients taking oral corticosteroids
Age over 50	Patients with active malignancies
Patients who agreed to participate in the trial and signed the informed consent form	Chronic metabolic diseases (parathyroid disorders, chronic renal failure)
Serum calcium is greater than 8–11 mg per deciliter	Patients with polytrauma
Unilateral intertrochanteric femur fractures	Previous use of ZOL within one year of hip fracture

Informed consent was obtained from all study subjects. After being admitted to the hospital, a thorough history was obtained from the patients and/or their attendants to ascertain the mode of injury and the magnitude of the trauma. All patients underwent in-depth examinations. Their overall health, systemic illnesses, and resulting injuries were noted. The Boyd and Griffith (BG) classification and the Arbeitsgemeinschaft für Osteosynthesefragen (AO) classification were used to classify all of the fractures, of which BG1 was considered stable. BG2, BG3, and BG4 were unstable fractures. All patients were treated with osteosynthesis with the proximal femoral nail (PFN).

Sample size

From a previous study by Li et al. [12], we observed that cases in the treatment group healed more quickly than those in the control group, i.e., 13 ± 3.2 weeks vs. 15 ± 4.6 weeks with a p-value of 0.02. Considering this value as a reference, with a power of 80% and a 95% confidence interval, the calculated sample size is 68 in each group, i.e., the total sample size is 136 [12].

Patients were classified into two groups based on simple randomization (online software [13] http://cafe.naver.com/easy2know/6427), namely Group T, the test group, in whom a single dose of ZOL injection (4 mg) was given on postoperative day 3 or before discharge, and Group C, the control group, where intervention was given after fracture healing. Follow-up of the patients was done at one, three, and six months after surgery. During each visit, patients were evaluated using the Radiographic Union Score for Hip (RUSH) score, the Singh Index (SI) for radiological outcomes, and the Harris Hip Score (HHS) and Visual Analog Scale (VAS) for functional outcomes. Clinically, when there was no tenderness or subjective symptoms, and radiographically, when the fracture line was not evident in the anteroposterior and lateral radiographs, the fracture was considered to be united. The HHS consists of five main domains: pain (0-44 points), function (0-33 points), functional activities (0-14 points), deformity (0-4 points), and range of movement (0-5 points). The maximum score is 100. It is filled out by both the patient and the clinician [14].

The RUSH score, which ranges from 10 to 30 points, is mostly composed of the cortical bridge index (4-12 points), visibility of the cortical fracture line (4-12 points), and the two trabecular indices, ranging from one to three for trabecular consolidation and trabecular fracture line visibility. Complete union is defined as a total RUSH score of 18 or higher [15].

Statistical analysis

Data collected were analyzed using IBM SPSS Statistics for Windows, Version 20.0 (Released 2011; IBM Corp; Armonk, New York, United States). Quantitative data were presented as mean, median, and SD, whereas qualitative data were presented as frequency and percentages. Based on the type and distribution of the data, the appropriate statistical tests were applied. A p-value <0.05 was taken as the level of significance. The Shapiro-Wilk test was used to determine data normality. A Mann-Whitney U test and an independent t-test were used.

## Results

A total of 252 patients with intertrochanteric femur fractures were presented to the hospital during the course of the trial. After applying strict inclusion and exclusion criteria, we included 136 patients in our study group. The overall mean age was 73.25 years; in Group T, it was 72.5 ± 11.9 years, and in Group C, it was 73.7 ± 11.8 years, with a high preponderance in the age group 71-80 years, with 44 patients that accounted for 32.3% of the study subjects. Out of 136 patients, 69 (51%) were males and 67 (49%) were females. Seventy-six (55.9%) patients had a fracture on the right side and 60 (44.1%) on the left side. Fractures were classified as per BG classification; the most common type of fracture in Group T was BG2 (64.7%), and in Group C, BG2 (69.1%). The length of stay in the hospital was 9.21 (3-21) days. It varied among patients due to factors like the availability of the operating room and the comorbid conditions of the patients (Table 2).

**Table 2:** Demographic characteristics. Group T: test group; Group C: control group; M/F: male/female; BG: Boyd and Griffith classification; PFN: proximal femoral nail. *Statistically significant at p-value <0.05.

Variables	Group T	Group C	p-value
Age (years)	72.5 ± 11.9	73.7 ± 11.8	0.501
Gender (M/F)	36/32	33/35	0.607
Fracture to admission time (days)	3.0 ± 2.0	2.8 ± 2.1	0.523
Admission to surgery time (days)	3.9 ± 2.2	4.0 ± 2.1	0.783
Fracture to surgery time (days)	6.9 ± 2.7	6.8 ± 2.7	0.646
Length of stay (days)	9.2 ± 3.3	9.2 ± 3.7	0.815
Side of injury			
Left	27 (40%)	33 (49%)	0.300
Right	41 (60%)	35 (51%)	
Fracture classification			
BG2	44 (65%)	47 (69%)	0.860
BG3	15 (22%)	13 (19%)	
BG4	9 (13%)	8 (12%)	
Type of PFN			
Long	56 (82%)	52 (76%)	0.803
Short	12 (18%)	16 (23%)	

The average fracture healing time in Group T was 12.2 ± 3.6 weeks, while it was 13.0 ± 2.8 weeks in Group C. There was no statistically significant difference in fracture union time between the groups (p-value of 0.56) (Table 3).

**Table 3:** Clinical outcomes. Group T: test group; Group C: control group. *Statistically significant at the 0.05 level, i.e., p-value <0.05.

Clinical outcomes	Group T	Group C	p-value
Time to union (mean - weeks)	12.2 ± 3.6	13.0 ± 2.8	0.56
Nonunion	1 (1.5%)	2 (2.9%)	0.774
Delayed union	4 (5.9%)	2 (3%)	0.360
Implant failure	1 (1.5%)	0	0.101
Varus angulation	4 (5.9%)	3 (4.4%)	0.128

Functional outcomes, including HHS, were found to be better in Group T than in Group C (p < 0.005). The median HHS at one, three, and six months were 42.7, 63.4, and 83.2, respectively. At six months, there was no statistically significant difference in HHS seen among the groups. Overall, 80% and 20% of patients, respectively, had excellent to good and fair to poor results, whereas 83.8% and 75% of patients in Group T and Group C, respectively, had excellent to good outcomes (Table 4).

**Table 4:** Harris Hip Score. Group T: test group; Group C: control group. ^†^Statistically significant at the 0.05 level, i.e., p-value <0.05. ^**^Statistically significant at the 0.001 level, i.e., p-value <0.001. *Statistically significant at the 0.01 level, i.e., p-value <0.01.

Variables	Group T (N = 68)		Group C (N = 68)		p-value
	Mean	SD	Mean	SD	
HHS-1 Month	42.8	1.5	42.3	1.6	0.029^†^
HHS-3 Month	62.6	4.7	61.6	3.2	<0.001^**^
HHS-6 Month	81.8	7.6	80.3	7.1	0.004^*^

In our study, radiological healing was seen in 15.1% of patients at the first month of follow-up, in 76.7% of patients at the third month, and in 8.2% of patients at the sixth month. As a result, the majority of our patients had a radiologically defined union by the 12th week or third month.

At one, three, and six months, the median RUSH score was 14, 22, and 25, respectively. No significant difference was observed between the two groups with respect to the radiological union and the RUSH score (p = 0.204) (Table 5).

**Table 5:** Radiographic Union Score for Hip. Group T: test group; Group C: control group; RUSH: Radiographic Union Score for Hip. *Statistically significant at the 0.05 level, i.e., p-value <0.05.

Variables	Group T (N = 68)		Group C (N = 68)		p-value
	Mean	SD	Mean	SD	
RUSH-1Month	13.5	1.8	13.9	1.8	0.103
RUSH-3Month	20.7	2.9	21.2	3.8	0.204
RUSH-6Month	23.6	3.2	24.3	4.3	0.068

At one, three, and six months, the mean SI for Group T and Group C was 3.8, 3.8, and 3.9 and 3.9, 4.0, and 4.0, respectively ( p = 0.297) (Table 6).

**Table 6:** Singh Index. Group T: test group; Group C: control group; M: month. *Statistically significant at the 0.05 level, i.e., p-value <0.05.

Variables	Group T (N = 68)		Group C (N = 68)		p-value
	Mean	SD	Mean	SD	
SINGH INDEX-1M	3.8	0.7	3.9	0.8	0.370
SINGH INDEX-3M	3.8	0.7	4.0	0.9	0.279
SINGH INDEX-6M	3.9	0.7	4.0	0.9	0.335

At one, three, and six months, the mean VAS for Group T and Group C was 4.4, 2.0, and 0.6 and 4.2, 1.9, and 0.6, respectively (p = 0.442) (Table 7).

**Table 7:** Visual Analog Scale. Group T: test group; Group T: control group; VAS: Visual Analog Scale. *Statistically significant at the 0.05 level, i.e., p-value <0.05.

Variables	Group T (N = 68)		Group C (N = 68)		p-value
	Mean	SD	Mean	SD	
VAS-1MONTH	4.4	0.7	4.2	0.8	0.122
VAS-3MONTH	2.0	0.7	1.9	0.7	0.442
VAS-6MONTH	0.6	0.7	0.6	0.7	0.700

**Figure 2:**
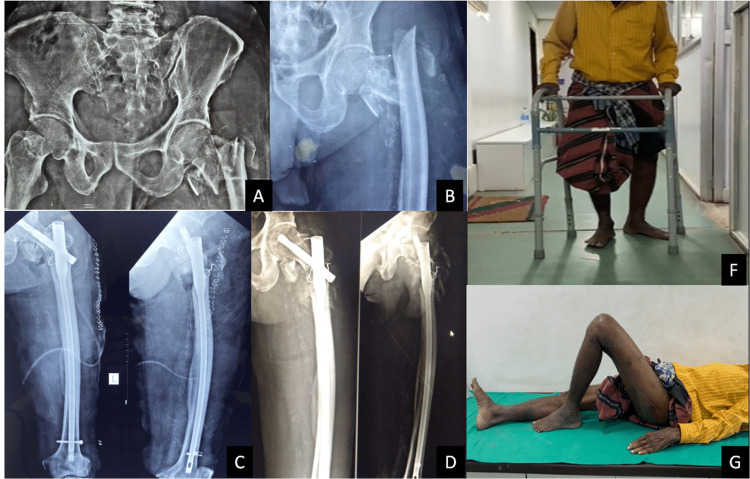
Preoperative radiographs (A, B), immediate postoperative radiograph (C), showing callus formation at six weeks (D), and six weeks of follow-up (E, F).

**Figure 3:**
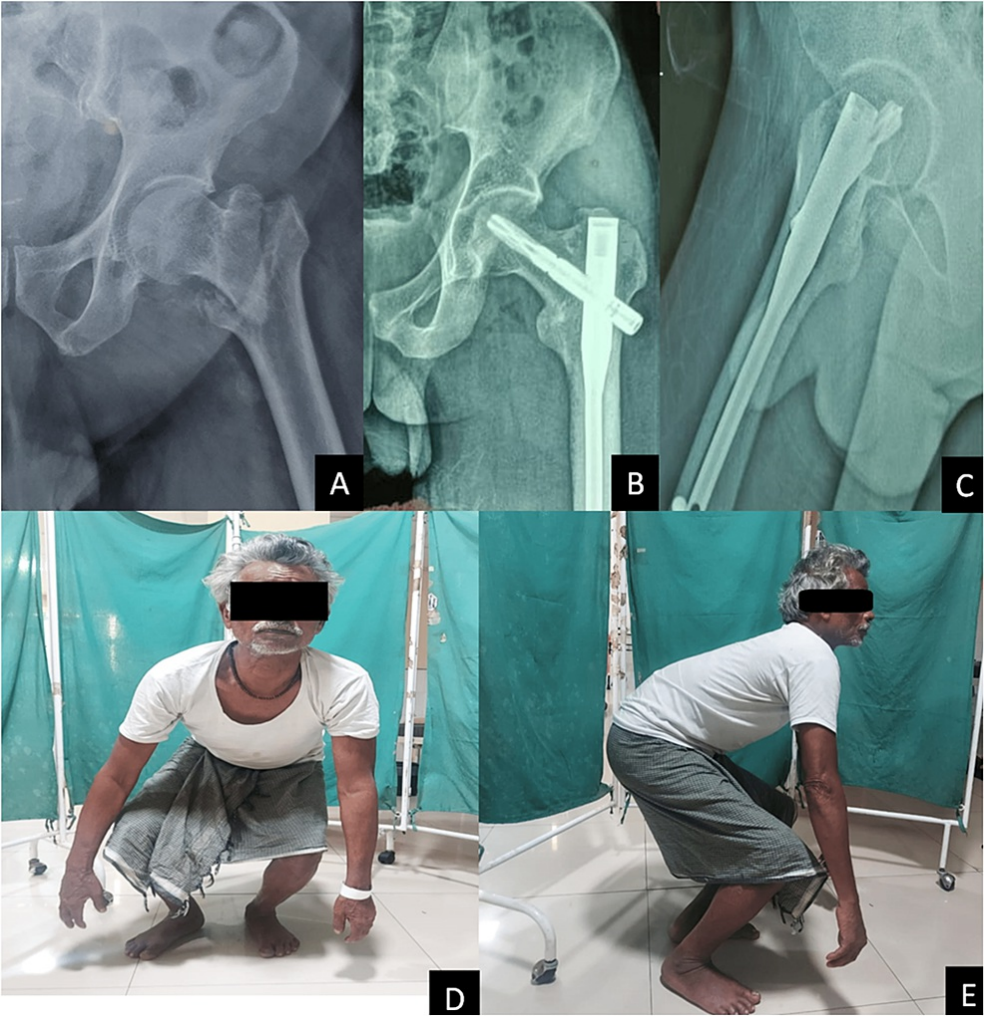
Preoperative anteroposterior (AP) view showing an intertrochanteric fracture (A), follow-up radiographs at 3 months postoperatively showing satisfactory fracture healing (B, C), and squatting without any discomfort (D, E).

 A few cases are illustrated in Figures 2 and 3.

## Discussion

Osteoporotic fractures are caused by trivial trauma, such as a fall from a standing height or less, or trauma that in a healthy person would not cause a fracture [16]. BPs play a crucial role in the treatment and prevention of osteoporosis, as they reduce the overall risk of fracture among osteoporosis patients and have a long-lasting beneficial impact [17].

Daily BP regimens are not chosen because they are less likely to result in long-term compliance than weekly regimens [18]. Once a month, BPs improve compliance by 47% at six months when compared to a once-weekly regimen [19], whereas administering an intravenous BP (ibandronate) once every three months results in higher adherence [20]. There are very little data available on the compliance of ZOL. A study conducted in our hospital between 2019 and 2021 on osteoporotic intertrochanteric femur fractures found that only 16% of patients had received osteoporotic treatment. These data show that compliance with BPs is very low in a tertiary care center.

The administration of ZOL during a hospital stay for the treatment of a fracture is opportunistic. If this opportunity is missed, the patients will need readmission to the hospital later for a ZOL injection. The logistics of ZOL administration, coupled with surgeons' fear of ZOL retarding bone union, can be a barrier to compliance for osteoporosis management.

Kates and Ackert-Bicknell [10], based on a recent report, found that ZOL exerts a negative effect on bone healing. According to Rozental et al., BP users with distal radius fractures report slow fracture healing. [21]. The BP group's mean time to union was 55 (17) days as compared to the control group's 49 (14) days. Additionally, as per Solomon et al. [9], BP usage during the post-fracture period was associated with a higher risk of nonunion (odds ratio: 2.37, 95% confidence interval: 1.13-4.96). Edwards et al. analyzed the FDA Adverse Event Reporting System database and discovered that using BPs increased the risk of delayed or nonunion femoral fracture by up to 26% [22]. For acute osteoporotic spinal fractures, Ha et al. concluded that BP therapy should be suspended during the healing process [23].

However, Fleisch conducted an animal experiment and found that BP had no negative or positive effect on callus size or fracture healing [24]. According to Lyles et al. [25], administering a yearly ZOL injection within 90 days of a pertrochanteric femur fracture surgery is associated with a lower rate of new clinical fractures and a higher survival rate. According to Gong et al., early initiation of BP therapy for patients with surgically treated osteoporotic distal radial fractures did not affect radiographic or functional outcomes or fracture healing [26]. ZOL dramatically enhanced callus volume and mechanical strength after a single dose, according to research by Amanat et al. [27]. Jalan et al. studied 123 intertrochanteric femur fractures in a randomized controlled trial (RCT) and discovered that "early administration of ZOL did not impair fracture healing in patients who underwent IT fracture fixation" [28]. In their study, Kim et al. observed that the timing of BP medication after pertrochanteric fracture surgery in osteoporotic patients had no influence on the incidence of complications or the radiologically determined fracture healing [29].

In a study of 114 patients with intertrochanteric femoral fractures, Cengiz et al. [30] found that the mean HHS in the treatment (zoledronate) and control groups was found to be 81.93 and 72.9, respectively, with the treatment group doing significantly better (p < 0.005). At a one-year follow-up, they found that the treatment group's bone mineral density (BMD) increase was significantly higher, and it was concluded that ZOL after intertrochanteric fracture surgery was found to help lower mortality, enhance the quality of life, and reduce bone pain in older patients [30]. In a study, Li et al. divided 60 osteoporotic patients with intertrochanteric fractures treated surgically with the PFN (12). However, no significant differences were found between the two groups with respect to HHS, and the mean duration of healing was 13 ± 3.2 and 15 ± 4.6 weeks, respectively, for the treatment (ZOL) and control groups (solely calcium and vitamin) (12).

In our research, we studied whether the use of ZOL in the early postoperative period affected the radiographic and clinical healing of fragility intertrochanteric fractures managed surgically in the eastern Indian population. Our study on 136 patients in an RCT demonstrates that the functional outcomes, including the HHS, were found to be better in Group T than in Group C (p < 0.005). It can significantly alleviate bone pain, enhance the quality of life, increase BMD, and prevent new fractures. We found similar RUSH scores and Singh Index at one, three, and six months after injury in both groups, implying no negative effect of ZOL on fracture healing (p > 0.005). Additionally, administering ZOL did not appear to have any detrimental effects on union in our cohort of patients.

The average age of the participants in our study was 73.25 years, which was comparable to the three groups in Kim et al.'s study [29], which had mean ages of 75 years for Group A, 75.3 years for Group B, and 78.1 years for Group C. Kim et al. [29] concluded that all fractures in both the intervention and nonintervention groups were joined at 20 and 24 weeks, respectively.

There was a delay in union in four cases in Group T and two cases in Group C. However, they were all united for six months. According to mortality data, one patient died in Group C, and none died in Group T. One patient had implant failure (the "Z effect") in Group T, and one patient in both groups had nonunion requiring revision surgery. All patients with fragility intertrochanteric fractures received intravenous ZOL for the management of osteoporosis. However, no statistical evidence of a correlation was found between age, gender, and time to union, and the p-values of all the variables were not significant. This study demonstrates that orthopedic surgeons should not miss the golden opportunity to administer ZOL in fragility fractures in the early postoperative period to achieve 100% compliance with osteoporosis management. This pragmatic approach will overcome one of the barriers to evidence-based practice for the management of fragility fractures in India.

Limitations

Single-Center Clinical Trial Study

To establish a correlation between age, gender, and time taken to fracture union, a large sample size will be required.

## Conclusions

This study demonstrates that postoperative intravenous ZOL therapy does not retard fracture healing. Osteoporosis management is frequently neglected because of a surgeon's fear of ZOL retarding fracture healing and a lack of awareness among patients, resulting in low compliance. Thus, in these circumstances, opportunistic administration of ZOL postoperatively can be beneficial and will increase compliance for osteoporosis management and prevention to 100%. This research will help to formulate the policy regarding the use of ZOL and will ensure up to 100% compliance for osteoporosis treatment. It is conceivable that the once-yearly application would potentially guarantee adherence for at least 12 months and therapeutic coverage for approximately 15 months.
